# Exploring #HealthLiteracy on LinkedIn: a first look at health hashtag engagement in a professional social media context

**DOI:** 10.3389/fdgth.2025.1622983

**Published:** 2025-07-04

**Authors:** Siva Sai Chandragiri, Bikash Baral, Fabian Peter Hammerle, Olena Litvinova, Elisa Opriessnig, Maima Matin, Himel Mondal, Michel-Edwar Mickael, Maria Kletecka-Pulker, Atanas G. Atanasov, Thomas Wochele-Thoma

**Affiliations:** ^1^ Department of Pathology, University of Oklahoma Health Sciences Center, Oklahoma City, OK, United States; ^2^ Organismal and Evolutionary Biology Research Programme, Faculty of Biological and Environmental Sciences, University of Helsinki, Helsinki, Finland; ^3^ Ludwig Boltzmann Institute Digital Health and Patient Safety, Medical University of Vienna, Vienna, Austria; ^4^ Department of Anaesthesia, Intensive Care Medicine and Pain Medicine, Division of General Anaesthesia and Intensive Care Medicine, Medical University of Vienna, Vienna, Austria; ^5^ Department of Management, Marketing and Quality Assurance in Pharmacy, National University of Pharmacy of the Ministry of Health of Ukraine, Kharkiv, Ukraine; ^6^ Medical University of Graz, Graz, Austria; ^7^ Institute of Genetics and Animal Biotechnology of the Polish Academy of Sciences, Magdalenka, Poland; ^8^ Department of Physiology, All India Institute of Medical Sciences, Deoghar, India; ^9^ Institute for Ethics and Law in Medicine, University of Vienna, Vienna, Austria

**Keywords:** LinkedIn, health literacy, hashtag analysis, social media, public health communication, digital engagement

## Abstract

LinkedIn, despite its large and professionally credentialed user base, remains an underexplored platform for digital health communication, unlike X (formerly Twitter), which has been widely studied for health-related hashtag trends. Health literacy, a key determinant of public health, is increasingly promoted through social media hashtags such as #HealthLiteracy. However, to date, no studies have systematically examined how this hashtag is used on LinkedIn. This study aimed to analyze the use of #HealthLiteracy on LinkedIn, identify thematic patterns in post content, and evaluate user engagement trends, with comparisons to prior X-based research. A one-week retrospective dataset of #HealthLiteracy posts was collected using the SingleFile browser extension. The content was cleaned and analyzed using RStudio with standard text mining packages. Word frequencies, co-occurring hashtags, and engagement metrics (likes, comments, reposts) were extracted, and a chi-square goodness-of-fit test was performed to assess engagement distribution. A total of 370 posts with 3,174 engagements were analyzed. The most frequent co-occurring hashtags included #agedcare, #residentialagedcare, and #healthquality, indicating a focus on institutional eldercare. The most common words, care, aged, solutions, and transition reinforced this thematic alignment. Engagement was primarily passive, with reactions far outnumbering comments and reposts. This study establishes a proof-of-concept for LinkedIn-based hashtag analysis in health research. LinkedIn demonstrates strong potential for targeted dissemination of health literacy content to professionals and policymakers, although engagement strategies may need to be tailored to the platform's predominantly passive interaction culture.

## 1 Introduction

With the global expansion of internet access and the widespread use of smartphones, social media platforms like Facebook, X, LinkedIn, and BlueSky have become central tools for public health communication and knowledge dissemination (1–3). Among these, Twitter (now X) has been extensively studied for its role in shaping health discourse through user-generated content, short-form messaging, and hashtag networks (4–7). Health-related hashtag analyses on X have provided valuable insights into information trends, public sentiment, misinformation, and digital health interventions (7–10). However, while X's open and conversational format has made it a rich site for research, other platforms particularly those with a more professional user base remain underexplored.

LinkedIn, a platform with over 900 million users globally, primarily targets professionals across diverse fields, including healthcare, academia, and policy (11). Unlike X, LinkedIn encourages longer-form content, professional validation, and topic-specific engagement within a more credentialed user community. These platform differences suggest that LinkedIn may foster more credible, nuanced, and professionally vetted health communication (12). Yet, despite these affordances, LinkedIn has not been systematically studied for its potential in health information dissemination particularly through hashtag use analysis.

Health literacy, defined as the capacity to obtain, process, and understand basic health information to make informed decisions, is a critical determinant of public health outcomes (13, 14). The digital era has expanded the concept of health literacy to include the ability to navigate online content, assess credibility, and engage with multimedia resources referred to as *digital health literacy* (15). Social media hashtags such as #HealthLiteracy have emerged as tools to cluster related content and promote visibility around these themes. While research has shown that X hashtags like #HealthLiteracy or #PublicHealth can drive significant engagement and even influence Altmetric scores (16–18), the use of such hashtags on LinkedIn remains undocumented.

On this background, the present study asks: How is the hashtag #HealthLiteracy being used on LinkedIn, and what themes and engagement patterns characterize these posts? Thereby, this study offers a novel contribution by conducting the first systematic analysis of #HealthLiteracy posts on LinkedIn, aiming to explore how this professional networking platform facilitates discourse and dissemination of health-related knowledge. The analysis focuses on patterns in post content, user engagement metrics (likes, comments, shares), and the use of co-occurring hashtags, based on data extracted during a one-week observation period. Furthermore, the study compares LinkedIn engagement trends with those observed in prior X-based hashtag analyses to highlight the unique affordances of professional social networking in public health communication. Ultimately, this work serves as a proof-of-concept for LinkedIn-based hashtag research and positions the platform as an underutilized yet promising space for digital health analysis.

## 2 Methods

### 2.1 Data collection

In March 2024, we conducted a retrospective one-week analysis of posts containing the hashtag #HealthLiteracy on LinkedIn. Due to the absence of a public Application Programming Interface (API) for LinkedIn and limitations on data accessibility, an adapted extraction method was implemented using the browser extension SingleFile (available for Firefox, Chrome, and Edge) (19). This extension allows for the complete export of web pages rendered as single HTML files for offline analysis.

The data collection procedure involved the following steps: (i) accessing LinkedIn's keyword search page for #HealthLiteracy using an existing LinkedIn profile, (ii) continuously down-scrolling and clicking “Show more results” to load additional posts until no further content was displayed, and (iii) exporting the entire loaded page using SingleFile. This approach allowed access to realistic engagement patterns and established professional networks, although it may introduce some algorithmic personalization bias. To mitigate this, research was refreshed at regular intervals throughout the analysis period to ensure maximal data capture and minimize potential time-based or profile-based sampling bias. All resulting HTML files were then compiled, and duplicate posts across exports were identified and removed through parsing procedures using regular expressions.

### 2.2 Data processing and analysis

The data collected were analyzed using RStudio (20), utilizing a range of packages including dplyr (21), stringr (21), tm (22), tidytext (22, 23), and officer (24). Preprocessing steps were implemented to clean and structure the text content. These included the removal of punctuation, numbers, emojis, and stop words (e.g., personal pronouns, adjectives, conjunctions) using the gsub and removeWords functions to enhance the quality of semantic analysis. To ensure consistency, all text data were converted to lowercase, and duplicated word variants were consolidated using functions from the tidytext package.

Hashtag terms were parsed separately to allow co-occurrence analysis, while terms beginning with the hashtag symbol (#) were excluded from the main word frequency count using regular expressions via the stringr package. A term-document matrix was generated to quantify the frequency of each term across all posts. From this matrix, the Top 200 most frequently used words and a comprehensive list of co-occurring hashtags were extracted to identify dominant themes and patterns in professional discourse surrounding health literacy.

Engagement metrics likes, comments, and shares were extracted from each post where available, allowing for a basic engagement trend analysis. Due to LinkedIn's technical constraints, data collection was limited to content visible within the one-week window preceding the extraction date, and the absence of real-time API access prevented automated metadata retrieval.

No personal or private data were collected in this study; all data were obtained from publicly accessible LinkedIn posts. The analysis was conducted on aggregated, anonymized data (user identities were not disclosed), ensuring privacy. This study did not require institutional board approval as it analyzed public domain information.

### 2.3 Statistical analysis

Statistical analysis involved expressing user engagement metrics in both raw counts and proportions to evaluate interaction trends on LinkedIn posts containing the hashtag #HealthLiteracy. Engagement types of reactions, comments, and reposts were quantified across the dataset. A chi-square (χ^2^) goodness-of-fit test was applied to assess whether the distribution of these engagement types deviated significantly from an equal engagement pattern.

Frequencies were compared to determine the dominant mode of interaction, with proportions used to highlight the relative contribution of each engagement type to the overall total. Statistical testing was conducted using GraphPad Prism version 10.4.1 (GraphPad Software LLC, San Diego, CA, USA), with statistical significance defined at *p* < 0.05. Results indicated a significant imbalance in user interactions, with reactions occurring at much higher rates than comments and reposts (χ^2^ = 2,427, df = 2, *p* < 0.001), suggesting a trend toward passive engagement behavior on the platform.

## 3 Results

### 3.1 Overview of #HealthLiteracy post characteristics

The study revealed a total of 370 posts with 3,174 engagements (reactions, comments, and reposts) during the study period. The most frequently used unique words and hashtags were identified in LinkedIn posts containing the hashtag #HealthLiteracy during the one-week observation period. The analysis revealed strong thematic alignment with elder care and health education, as detailed below.

**Figure 1:**
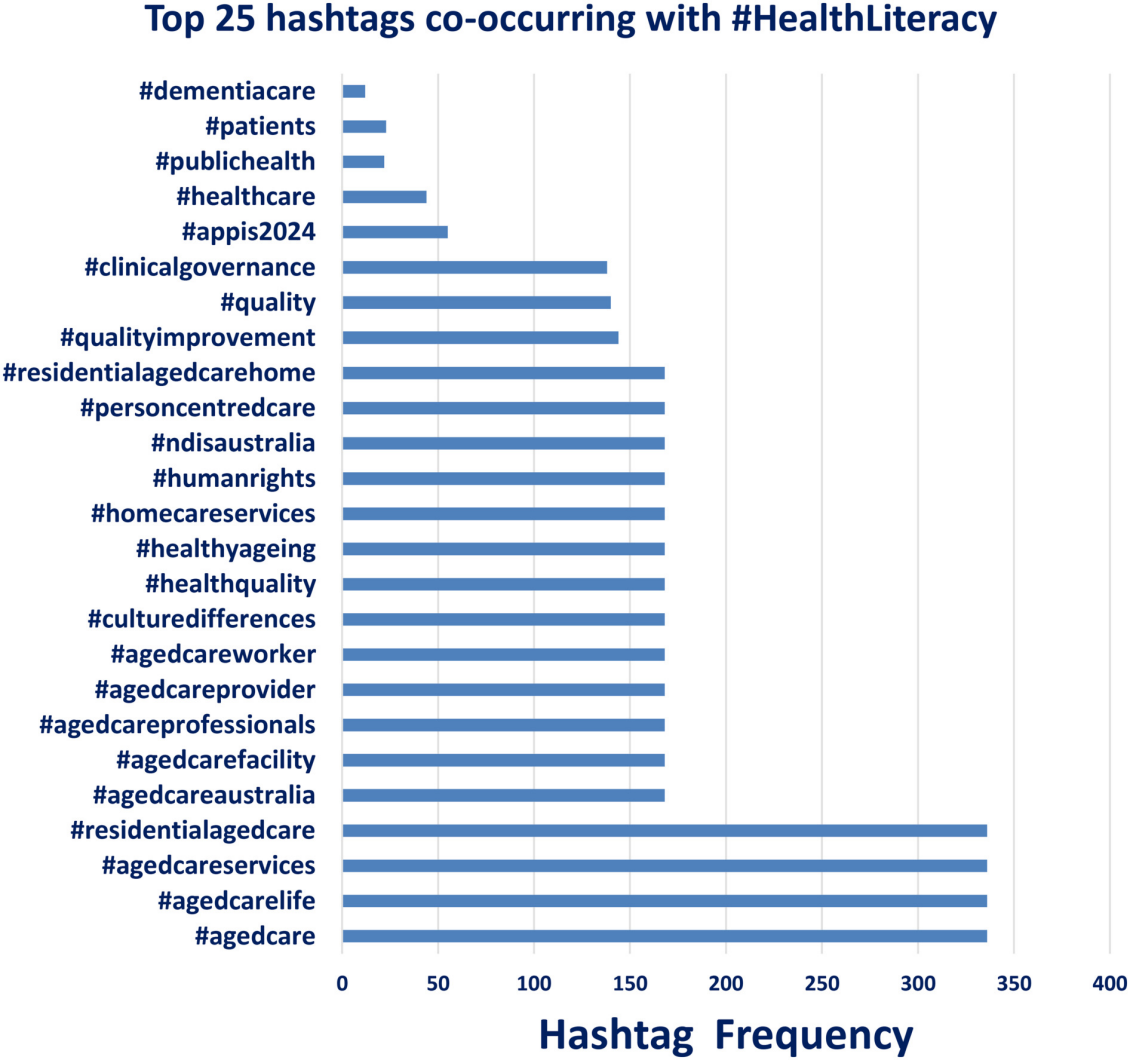
Top 25 hashtags co-occurring with #HealthLiteracy: Frequencies reflect the number of times each hashtag appeared in combination with #HealthLiteracy during the one-week observation period. The most frequent hashtags were strongly aligned with themes of aged care and professional healthcare service delivery.

Frequencies reflect the number of times each hashtag appeared in combination with #HealthLiteracy during the one-week observation period. The most frequent hashtags were strongly aligned with themes of aged care and professional healthcare service delivery (Figure 1).

Analysis of hashtag co-occurrence patterns revealed that content associated with #HealthLiteracy frequently intersects with themes related to aging, care services, and health equity. The most common co-occurring hashtags included #agedcare, #agedcarelife, and #agedcareservices, each appearing over 300 times in the dataset. Other prominent terms included #residentialagedcare, #agedcareaustralia, and #agedcareprofessionals, indicating a strong thematic linkage between health literacy discourse and professional aged care practices. Hashtags such as #healthquality, #homecareservices, and #personcentredcare also appeared frequently, further emphasizing a focus on care delivery standards and individualized patient experiences. Notably, public health-oriented hashtags like #publichealth and #healthcare were far less common in this LinkedIn-based dataset, suggesting that the professional conversation around #HealthLiteracy on this platform is more concentrated within eldercare and institutional care settings rather than broad health advocacy.

**Figure 2:**
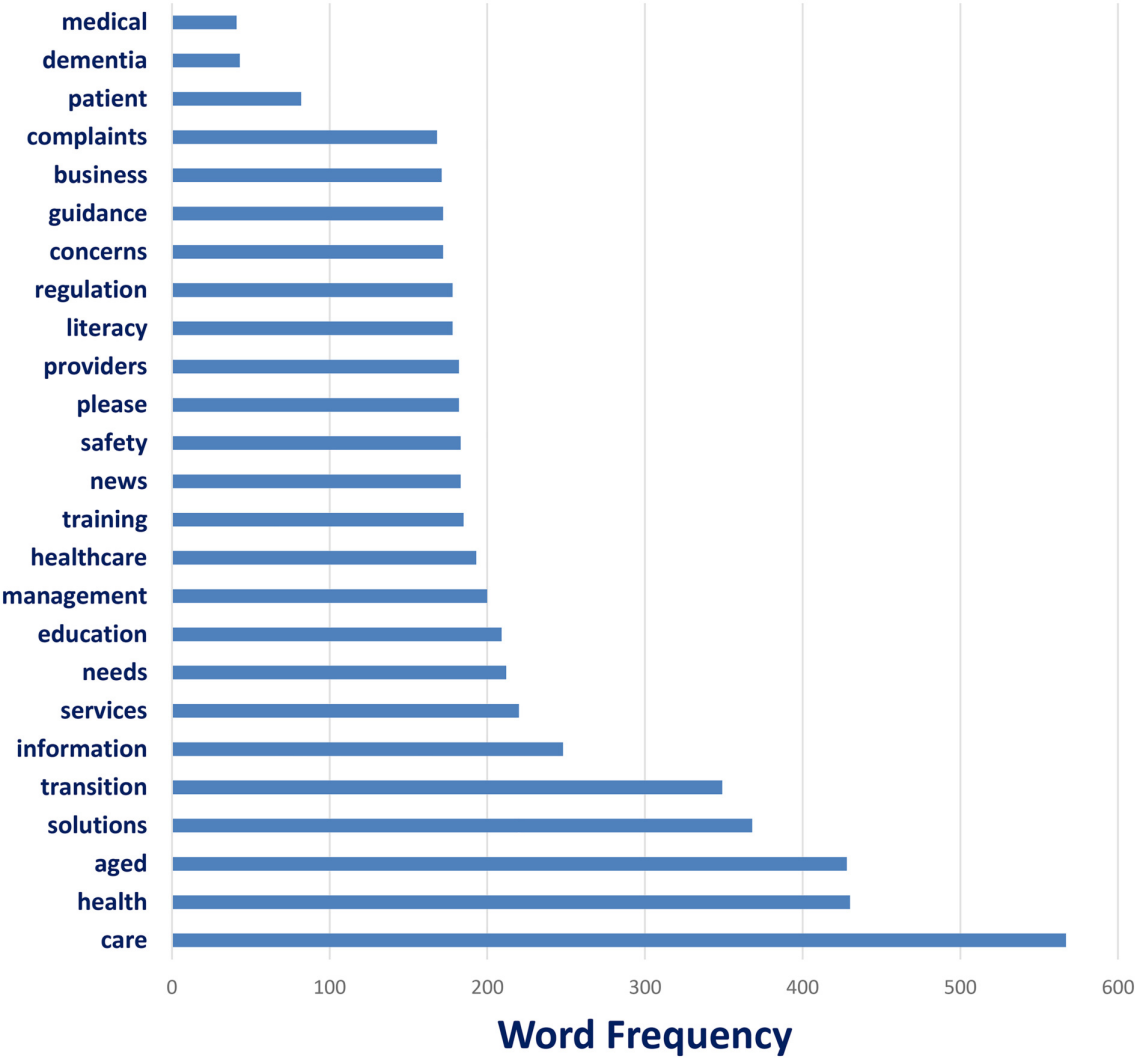
The top 25 words of the #HealthLiteracy posted: Word counts reflect the raw frequency of appearance in a one-week dataset of LinkedIn content, highlighting key themes around aged care, service transitions, and system-level communication in health contexts.

Word counts reflect the raw frequency of appearance in a one-week dataset of LinkedIn content, highlighting key themes around aged care, service transitions, and system-level communication in health contexts (Figure 2).

The content analysis of #HealthLiteracy posts on LinkedIn revealed recurring themes centered around elder care, health service delivery, and patient-centered transitions. As shown in Figure 2, the most frequently used words included care (*n* = 567), health (*n* = 430), aged (*n* = 428), solutions (*n* = 368), and transition (*n* = 332). These terms reflect a strong focus on navigating care models for aging populations, emphasizing systemic approaches and solution-oriented discussions. Other highly used words such as information, services, needs, and education underscore the importance of communication, accessibility, and empowerment within health literacy conversations. Terms like training, providers, and safety also point to the professional and regulatory dimensions of LinkedIn-based discourse. Interestingly, words traditionally associated with patient interaction such as medical and patient occurred far less frequently, suggesting that the dominant framing of #HealthLiteracy on LinkedIn may lean more toward institutional or administrative perspectives than direct patient engagement.

**Figure 3:**
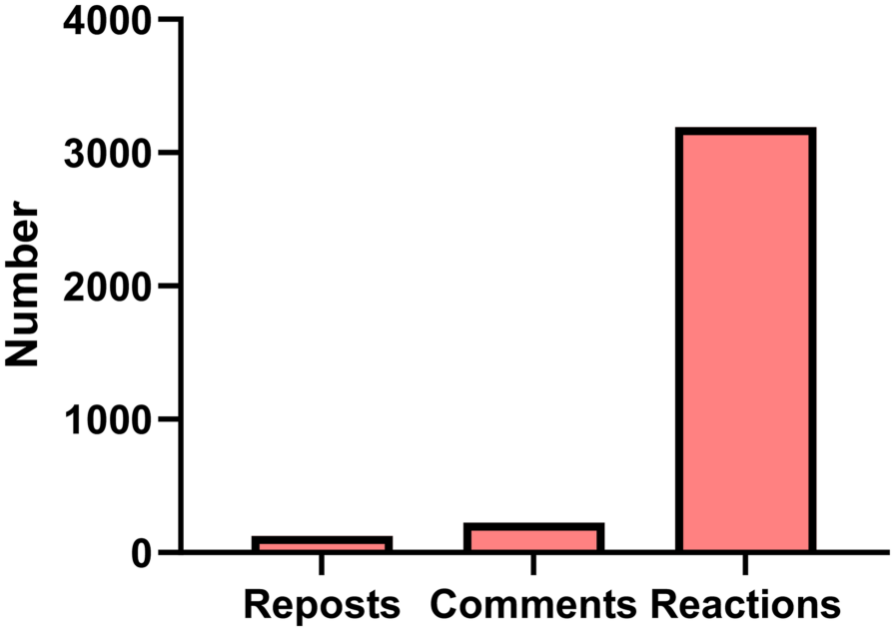
Engagement Patterns for #HealthLiteracy Posts on LinkedIn: Distribution of reactions (likes), comments, and reposts on LinkedIn posts containing the hashtag #HealthLiteracy during the one-week observation period. Reactions accounted for the majority of user engagement, indicating a predominantly passive interaction pattern.

Distribution of reactions (likes), comments, and reposts on LinkedIn posts containing the hashtag #HealthLiteracy during the one-week observation period. Reactions accounted for the majority of user engagement, indicating a predominantly passive interaction pattern (Figure 3).

Engagement analysis revealed a substantial imbalance in user interaction types with LinkedIn posts tagged with #HealthLiteracy. As depicted in Figure 3, reactions (likes) accounted for the vast majority of interactions (*n* = 3,193), followed by comments (*n* = 225) and reposts (*n* = 126). A chi-square goodness-of-fit test was conducted to determine whether these observed frequencies significantly differed from an equal distribution. The results indicated a statistically significant difference in engagement types (χ^2^ = 2,427, df = 2, *p* < 0.001), confirming that users were significantly more likely to react to content than to comment on or repost it.

## 4 Discussion

This study offers the first systematic examination of health-related hashtag discourse on LinkedIn, establishing a proof-of-concept for the platform's use in health communication research. While social media platforms such as X have been widely studied for their role in health information dissemination, LinkedIn has remained largely overlooked despite its unique position as a professional networking site with a user base of healthcare workers, educators, policymakers, and organizational leaders. No prior studies have systematically examined health-related hashtags on LinkedIn, making this work an original and novel contribution to digital health communication literature.

The co-occurring hashtag analysis (Figure 1) revealed a strong thematic alignment between #HealthLiteracy and institutional care environments. Hashtags such as #agedcare, #residentialagedcare, and #agedcarelife were among the most frequent, suggesting that on LinkedIn, discussions about health literacy are highly embedded within eldercare, regulatory frameworks, and long-term service delivery. This focus stands in contrast to X-based studies, where #HealthLiteracy often intersects with broader advocacy or patient-centered initiatives (18, 25). The relatively low presence of public-facing hashtags like #publichealth or #healthcare on LinkedIn reinforces the idea that the platform is being used more for interprofessional discourse than public outreach.

The word frequency analysis (Figure 2) further supports this institutional framing. The most common terms care, health, aged, solutions, and transition emphasize systemic approaches to health literacy, including strategic management of care transitions and support services for aging populations. Meanwhile, the lower frequency of terms like medical or patient indicates that the conversation may prioritize structural and operational aspects of care rather than individual-level clinical encounters. These findings suggest that LinkedIn may serve as a valuable space for promoting organizational and policy-level approaches to health literacy, especially in domains such as workforce training, aged care reform, and care model innovation.

Notably, the co-occurring hashtag #dementia care and the keyword “dementia” also attracted particular attention from LinkedIn users. This seems warranted because in gerontological practice, cognitive impairment, particularly dementia, is often a clinically relevant consequence of the combined impact of chronic and age-associated diseases. According to WHO, dementia ranks seventh among the causes of death in the world and is the leading cause of disability in the older adult population (26). In 2019, its economic damage was estimated at $1.3 trillion, and by 2050 it is expected that about 152 million people around the world will live with dementia (27). These trends point to the need for systemic changes in approaches to prevention, diagnosis, and care of dementia patients and for improving medical literacy in this area. Along this line, the modeling conducted by Kingston A et al. indicates the need to adapt the health and social care system in the UK to an increase in the number of older people over 85 with dementia and concomitant diseases (28). Moreover, the study by Hendriks S et al. emphasizes the importance of raising awareness of dementia among young people and developing strategies for its early diagnosis and intervention, which can slow down the progression of the disease (29). Dementia has a serious psychological and physical impact on family members. Patient care is accompanied by prolonged stress, and a high level of health literacy among caregivers can significantly reduce stress and improve quality of life. Thus, the study by Xu XY et al. showed that social support reduced the risk of cardiovascular disease in relatives caring for dementia patients (30). In line with our findings, prior research on Facebook and Twitter confirms that the discussion of dementia is mainly carried out by the scientific and professional community (31). Thereby, the results of our LinkedIn analysis suggest that this platform can also be effectively used to increase the health literacy of the population in the dementia field, as well as to support caregivers and disseminate evidence-based information.

Engagement metrics (Figure 3) provide additional insights into how health literacy content performs on the platform. The vast majority of engagement came in the form of reactions (*n* = 3,193), far exceeding comments (*n* = 225) and reposts (*n* = 126). This pattern, confirmed by a statistically significant chi-square test outcomes (χ^2^ = 2,427, df = 2, *p* < 0.001), reflects a tendency toward passive engagement, where users acknowledge content but seldom amplify or discuss it. This may be due to the professional culture of LinkedIn, where users are more cautious about publicly commenting on sensitive topics or endorsing particular viewpoints (1, 32). The preference for low-effort interactions like “likes” suggests that while LinkedIn is a credible venue for exposure and visibility, it may be less suited to driving viral diffusion or interactive discourse without more targeted strategies.

## 5 Strengths, limitations, and future research directions

This study offers several key strengths. Most notably, it represents the first systematic analysis of health-related hashtag use on LinkedIn, making a novel contribution to the digital health communication literature. By applying a structured approach to examining post content, engagement metrics, and hashtag co-occurrence, the study provides a reproducible framework for future research exploring professional social media platforms.

However, there are limitations to consider. The dataset was restricted to a one-week retrospective snapshot, which, while sufficient for proof-of-concept, may not fully capture temporal variability or responses to real-time health events. Given the importance of public health issues, future work should address this constraint through longitudinal studies that monitor health hashtag trends over extended periods. Additionally, LinkedIn's restricted API access constrained the ability to automate data extraction and prevented continuous monitoring, limiting scalability. Furthermore, while prior research often uses Twitter as a reference platform, its declining popularity and shifting user base may reduce its utility as a consistent comparator in future cross-platform analyses.

Building on this foundation, future directions include tracking the evolution of hashtags like #HealthLiteracy in response to specific campaigns, seasonal health trends, or global policy shifts. Comparative cross-platform analyses (LinkedIn vs. Twitter/X vs. Facebook) could illuminate differences in reach, engagement, and discourse tone between professional and public audiences. Furthermore, incorporating advanced natural language processing (NLP) techniques such as sentiment analysis, misinformation detection, and topic modeling would deepen understanding of content quality and audience reception, strengthening the field's capacity to leverage LinkedIn for impactful, evidence-based health communication.

## 6 Key implications and conclusion

These findings underscore the untapped potential of LinkedIn for structured and strategic public health messaging. As a platform uniquely positioned to reach healthcare professionals, educators, and policymakers, LinkedIn offers an opportunity to communicate health literacy in ways that are credible, context-specific, and aligned with institutional practices. For example, hospital networks and academic medical centers could monitor trending hashtags like #HealthLiteracy to identify communication gaps and tailor professional development or patient education strategies accordingly. Public health educators might use co-occurring hashtag data to align campaign messages with language and themes that are already gaining traction within professional communities. Policy advocates can also leverage LinkedIn discussions to anticipate stakeholder concerns and frame proposals in terminology that resonates within regulated sectors. For public health campaigns aiming to affect change at the policy or systems level, LinkedIn may serve as a highly effective dissemination channel particularly when content is framed through a professional or regulatory lens. At the same time, the platform's culture of low-visibility engagement means that communicators must design content intentionally to prompt interaction such as by tagging relevant stakeholders, including multimedia assets, or aligning messages with ongoing industry themes.

In conclusion, this study not only introduces LinkedIn as a viable and underexplored site for digital health research but also sets the stage for future investigations into platform-specific communication strategies. By identifying thematic patterns, engagement trends, and content dynamics associated with #HealthLiteracy, it offers a foundational model for how LinkedIn can be leveraged in targeted, professional health literacy campaigns. These findings provide actionable insights that can inform institutional communication strategies, clinician-led health initiatives, and policy advocacy efforts. As digital health communication continues to evolve, including LinkedIn in social media research and practice toolkit will be essential to reaching and influencing professional audiences on a scale.

## Data Availability

The original contributions presented in the study are included in the article/Supplementary Material, further inquiries can be directed to the corresponding authors.
